# Iatrogenic lung overinflation resulting in pneumothorax and pneumoperitoneum during oxygen therapy after general anesthesia: A case report

**DOI:** 10.1097/MD.0000000000043410

**Published:** 2025-07-18

**Authors:** Jiyoung Kim, Jeong-Hee Kim, Hyeokjae Kwon

**Affiliations:** a Department of Nursing, Woosuk University, Wanju-gun, Jeonbuk, South Korea; b Department of Plastic and Reconstructive Surgery, College of Medicine, Chungnam National University, Daejeon, South Korea; c Department of Plastic and Reconstructive Surgery, Chungnam National University Hospital, Daejeon, South Korea.

**Keywords:** general anesthesia, lung overinflation, oxygen therapy, patient safety, pneumoperitoneum, pneumothorax, postoperative care

## Abstract

**Rationale:**

Oxygen therapy is a critical component of postoperative care, particularly after general anesthesia. Although generally safe, its improper administration can lead to serious complications. This report details an incident of lung overinflation during postoperative oxygen therapy that resulted in pneumothorax and pneumoperitoneum.

**Patient concerns:**

A 63-year-old female patient with tracheostomy underwent pressure sore reconstruction under general anesthesia. Postoperatively, the patient was transferred to the recovery room, and oxygen therapy was initiated. During oxygen administration, the anesthesiology nurse omitted the connection of a heat-moisture exchanger and thereby inadvertently administered excessive dry positive pressure ventilation. Subsequently, the patient developed acute respiratory distress.

**Diagnoses:**

Clinical examination revealed decreased bilateral breathing sounds and abdominal distension. Computed tomography confirmed bilateral pneumothorax and pneumoperitoneum.

**Interventions:**

Conservative management was chosen.

**Outcomes:**

The patient’s condition stabilized, and she was discharged after 3 weeks with no long-term complications.

**Lessons:**

This case highlights the importance of careful monitoring and adherence to appropriate techniques during postoperative oxygen therapy. Overinflation of the lungs can lead to life-threatening conditions such as pneumothorax and pneumoperitoneum. This incident highlights the need for rigorous training and vigilance among healthcare professionals to prevent such occurrences. Although oxygen therapy is essential for patients recovering from general anesthesia, this case illustrates the potential risks associated with improper administration. Awareness and preventive measures are crucial for avoiding similar adverse events and ensuring patient safety and optimal outcomes.

## 1. Introduction

Postoperative hypoxemia is a common respiratory complication that occurs after surgery owing to changes in pulmonary function and has a variety of causes.^[1]^ Hypoxemia, defined as a PaO_2_ of 60 mm Hg or less, requires attention, as it can present with nonspecific clinical signs such as changes in blood pressure and arrhythmias. Postoperative hypoxemia can delay recovery or cause major organ failure. Most patients admitted to the recovery room after general anesthesia should, therefore, receive oxygen to prevent hypoxemia.^[2]^

Oxygenation devices used in the recovery room include nasal prongs and reservoir masks for patients without a tracheostomy, and heat-moisture exchangers (HME) to prevent dryness for patients with a tracheostomy.^[3]^ If a nasal prong or reservoir mask is used in the recovery room after extubation, oxygenation can be provided relatively safely^[4]^; however, oxygenation through a tracheostomy requires special care.^[5]^

This case report presents an incident involving a 63-year-old female patient with a tracheostomy who developed pneumothorax, pneumoperitoneum, and subcutaneous emphysema due to lung overinflation during oxygen therapy after undergoing elective flap surgery for the reconstruction of a sacral pressure sore. This case illustrates the importance of following proper protocols and meticulous monitoring during postoperative oxygen therapy. By reporting this case, we hope to emphasize the need for thorough training of medical staff, increased monitoring, and awareness of the potential risks of postoperative oxygen therapy.

## 2. Case presentation

This study was approved by the Research Ethics Board of Chungnam National University Hospital (Daejeon, South Korea, IRB No. 2024-10-002), and informed consent was obtained from the participants.

A 63-year-old woman with a history of transverse myelitis involving the C2 spinal cord level, leading to tracheostomy, presented with a stage IV sacral pressure ulcer (7 × 6 cm) with exposed sacral bone, refractory to 12 weeks of conservative management, including wound debridement and negative pressure therapy. Surgical reconstruction was indicated due to high osteomyelitis risk and failed healing progression. She underwent flap reconstruction for a sacral pressure sore in the prone position. Under general anesthesia induced with propofol (2 mg/kg) and fentanyl (2 µg/kg), the airway was secured with a reinforced cuffed tracheostomy tube (ID 8.0 mm). Anesthesia was maintained with sevoflurane (1.5–2.0 MAC) in 40% oxygen and remifentanil infusion (0.1–0.2 µg/kg/min). Volume-controlled ventilation used a tidal volume of 6 mL/kg predicted body weight, positive end-expiratory pressure 5 cm H₂O, and peak inspiratory pressure ≤18 cm H₂O. The surgery lasted approximately 2 hours and was uneventful, with minimal blood loss and stable intraoperative hemodynamics. After surgery, she was transferred to the recovery room. Postoperatively, the patient remained alert (GCS: E_4 V_T M_NT, SECONDs score 7/8) and hemodynamically stable. Upon arrival at the recovery room, standard postoperative oxygen therapy protocol for tracheostomy patients was initiated. Passive oxygen therapy at 5 L/min was planned to apply via an HME connected to the tracheostomy tube. No positive pressure modalities were planned because baseline oxygenation was adequate. The nurse connected the patient to the standard monitoring and initiated oxygen therapy. However, owing to an oversight, oxygen was delivered directly to the patient’s tracheostomy tube without using a HME, implying that the pressure was not regulated, and heat and moisture were not exchanged. Because of the tracheostomy, the patient was unable to communicate directly, and 15 minutes after entering the recovery room, the monitoring device alarmed due to unstable vital signs, and the erroneous connection of the oxygen was discovered. The patient’s condition was immediately assessed. Her vital signs deteriorated as follows: blood pressure, 90/60 mm Hg; heart rate, 120 beats/min; respiratory rate, 28 breaths/min; and oxygen saturation, 88% despite high-flow oxygen.

On physical examination, the patient appeared anxious and diaphoretic. Chest auscultation revealed decreased breathing sounds on both sides. Palpation of the chest wall and neck revealed crepitus suggestive of subcutaneous emphysema.

**Figure 1.**
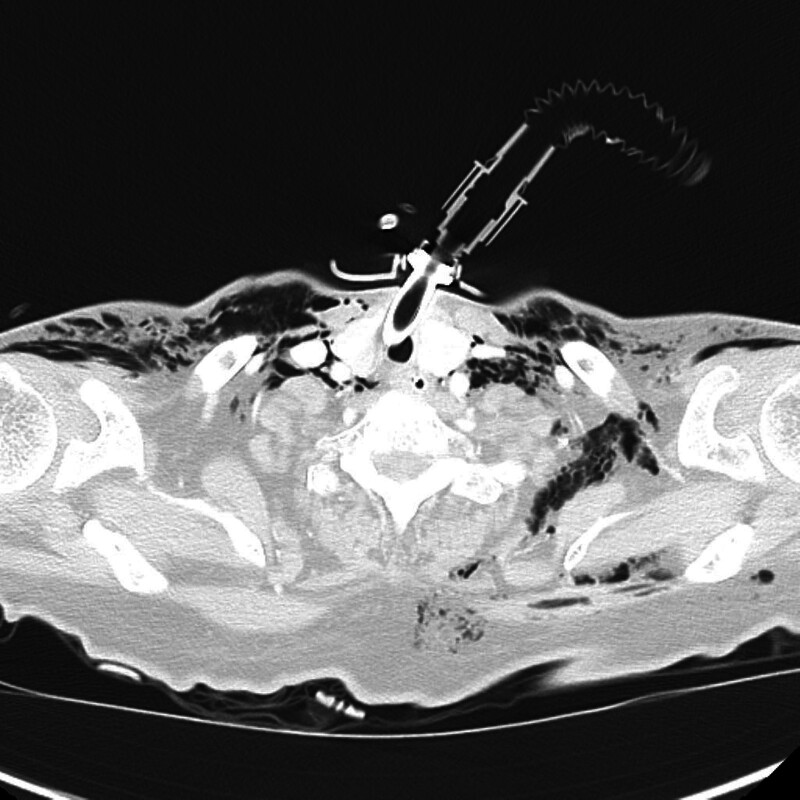
Axial chest CT image at the level of the thoracic inlet shows extensive subcutaneous emphysema. Air is noted dissecting through the fascial planes of the bilateral supraclavicular and paratracheal regions, extending into the posterior neck. A tracheostomy tube is visible in situ, and air locules surround the soft tissue structures, consistent with positive-pressure-induced barotrauma. CT = computed tomography.

**Figure 2.**
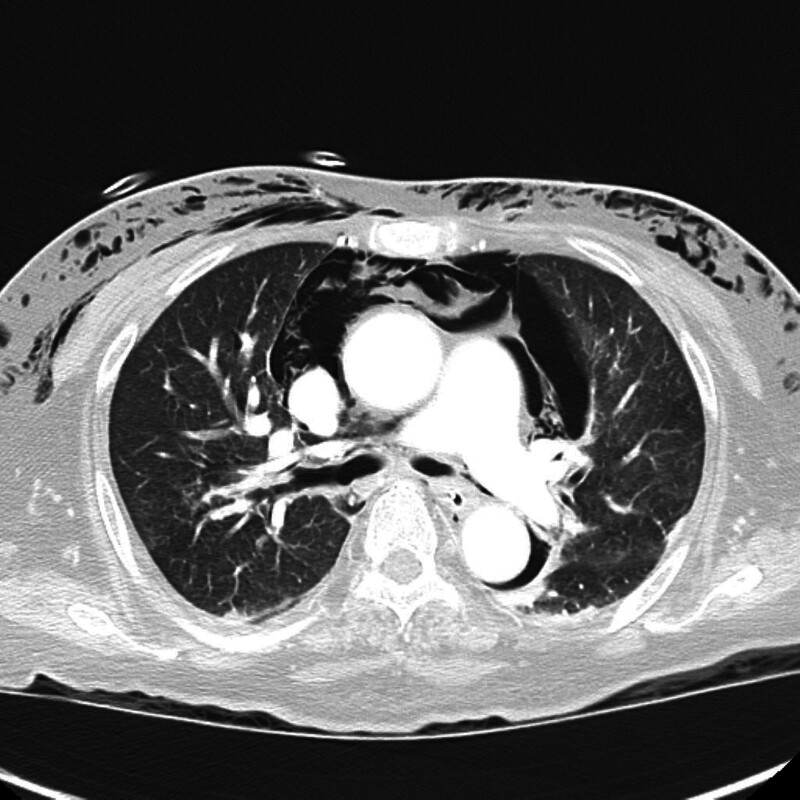
Axial chest CT at the mid-thoracic level reveals moderate bilateral pneumothorax. Both lungs show peripheral lucency, indicating free air in the pleural space, more prominent on the left side. The partially collapsed lungs exhibit peripheral atelectasis, and air density is visible in the subcutaneous tissues along the chest wall, indicating ongoing subcutaneous emphysema. CT = computed tomography.

**Figure 3.**
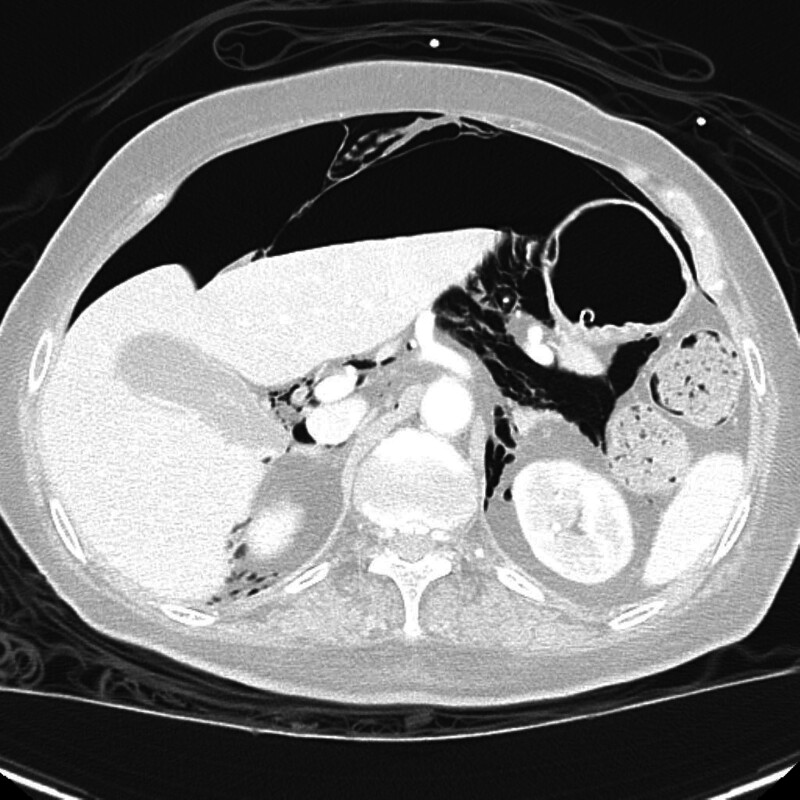
Axial CT at the lower thoracic level shows significant bilateral pneumothorax and diaphragmatic outline elevation. The left lung is markedly collapsed, and a large air pocket is visualized within the left pleural space. The diaphragm appears sharply outlined by subdiaphragmatic free air, supporting the presence of associated pneumoperitoneum. CT = computed tomography.

Given the patient’s clinical presentation, an urgent chest radiograph was obtained, which revealed bilateral pneumothorax and extensive subcutaneous emphysema. Computed tomography (CT) of the chest, abdomen, and pelvis was performed to evaluate the extent of air dissection. The CT findings were as follows (Figs. 1–3): moderate bilateral pneumothorax with partial lung collapse, extensive subcutaneous emphysema extending from the neck to the lower abdomen, and pneumoperitoneum with free air in the peritoneal cavity.

Despite significant radiological findings, the patient’s hemodynamic status began to stabilize following the correction of dry oxygen delivery with the application of an appropriate HME to her tracheostomy tube. After careful consideration of the patient’s overall clinical picture, including the comorbidities and risks associated with invasive procedures, the medical team opted for a conservative management approach. The patient was closely monitored in the intensive care unit with the following interventions: continuous pulse oximetry and cardiac monitoring, hourly vital sign checks, frequent physical examinations, high-flow humidified oxygen therapy through the tracheostomy with appropriate HME, serial chest X-rays to monitor the pneumothorax, and respiratory physiotherapy.

Over the next 48 hours, the patient’s condition gradually improved. Her oxygen requirements decreased, and repeat imaging showed progressive resolution of the pneumothorax, pneumoperitoneum, and subcutaneous emphysema. By postoperative day 5, the patient returned to baseline respiratory status, and a follow-up CT scan confirmed complete resolution of the air collection. The patient’s respiratory parameters are shown in Table 1.

**Table 1:** Respiratory parameters of the patient.

Timepoint	Respiratory rate (breaths/min)	SpO₂ (%)	FiO₂	Oxygen delivery method
Postoperative (15 min)	28	88	1.0	High-flow oxygen without HME (tracheostomy)
After HME applied	22	94	0.6	High-flow humidified oxygen with HME
Postoperative day 2	18	98	0.4	Tapered humidified oxygen

HME = heat-moisture exchanger.

**Figure 4.**
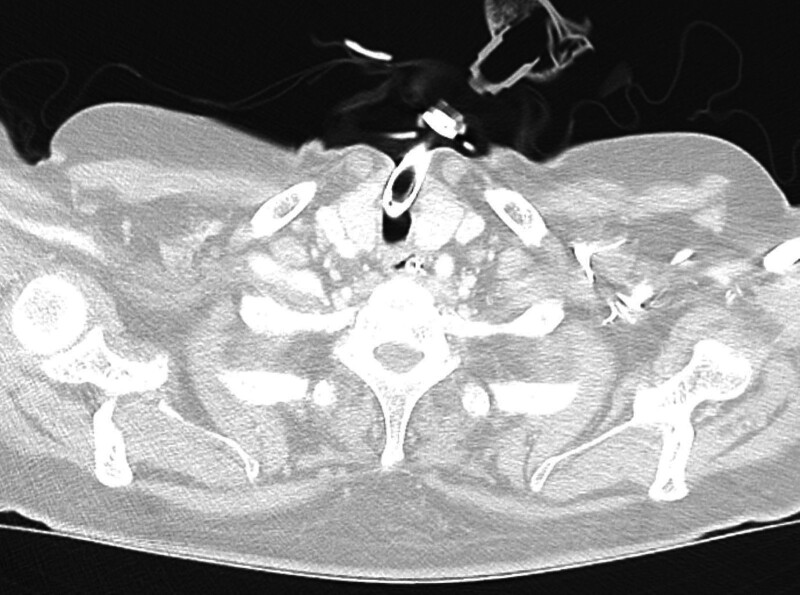
Follow-up axial CT scan at the thoracic inlet level shows resolution of subcutaneous emphysema. No residual air is visible within the soft tissues of the neck or chest wall. The tracheostomy tube remains in place without evidence of peritubal air leakage or re-accumulation. CT = computed tomography.

**Figure 5.**
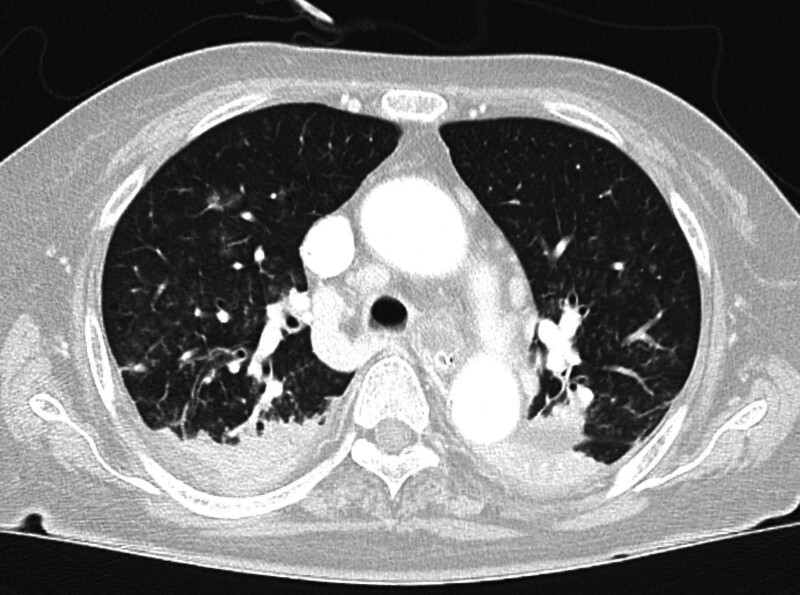
Follow-up axial chest CT at mid-thoracic level confirms resolution of pneumothorax. Both lungs are fully expanded with preserved architecture and no pleural air. Subcutaneous tissues appear normal, and the pleural reflections are intact, with no evidence of reaccumulation of air. CT = computed tomography.

**Figure 6.**
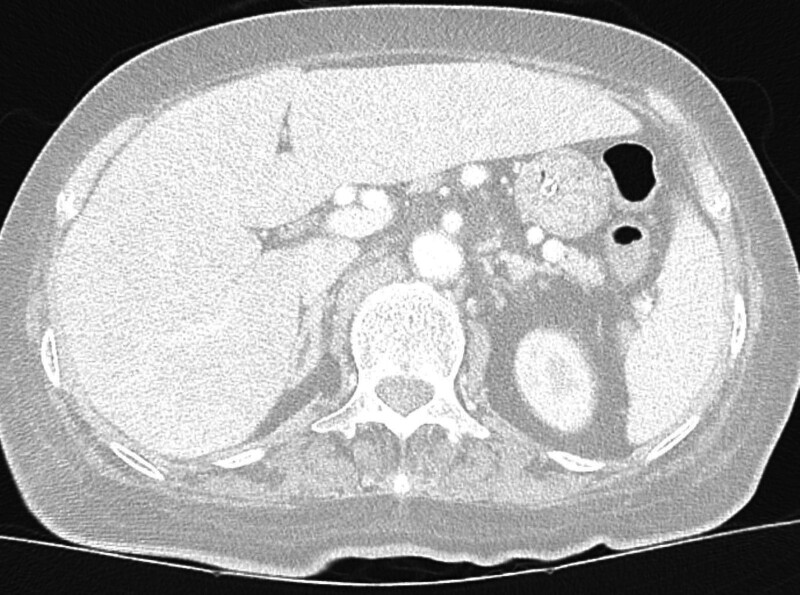
Follow-up abdominal CT scan demonstrates complete resolution of pneumoperitoneum. There is no visible free intraperitoneal air under the diaphragm or between bowel loops. The abdominal viscera, including the stomach and bowel, show normal distribution, and no signs of visceral perforation or inflammatory changes are noted. CT = computed tomography.

The patient was transferred to the ward on postoperative day 7 with no residual respiratory complications. At a 1-month follow-up, she remained stable with well-healed surgical sites and no recurrence of air-leak symptoms (Figs. 4–6).

This case highlights the potential for severe complications following improper airway management in tracheostomized patients and demonstrates that conservative management can be successful in carefully selected cases, even with extensive air dissection into multiple compartments.

## 3. Discussion

This patient experienced overinflation of the lungs with excessive positive pressure due to the direct application of oxygen through the tracheostomy tube without the use of an HME or pressure control. The direct oxygen delivery created unregulated positive pressure, leading to alveolar overdistension. Subsequent rupture caused air dissection through the mediastinum into the pleural space (pneumothorax) and retroperitoneal cavity (pneumoperitoneum).^[6]^ Although no definitive airway rupture was visualized on CT, the findings suggest alveolar rupture due to barotrauma, leading to air dissection through the mediastinum and into the subcutaneous tissue and peritoneal cavity.^[7]^ This case report also highlights a rare but potentially life-threatening complication arising from improper airway management in a tracheostomized patient. The inadvertent delivery of dry oxygen directly into the tracheostomy tube led to a cascade of events, resulting in pneumothorax, pneumoperitoneum, and subcutaneous emphysema. Postoperative oxygen therapy is essential, but in special circumstances, such as tracheostomy, inappropriate oxygenation can cause serious harm to the patient. Excessive positive pressure can cause overinflation, alveolar rupture, and air leakage into the pleural cavity, peritoneal cavity, and subcutaneous tissues, resulting in pneumothorax, pneumomediastinum, and subcutaneous emphysema. In this case, no invasive procedures were performed, such as chest tubes, and the patient recovered well. This indicates that clinical decisions can be based on the patient’s stability on a case-by-case basis to avoid invasive procedures.

This case highlights the need for increased training for healthcare providers, particularly in recognition of pulmonary overinflation and rapid response. Medical staff should be aware of this complication and take preventive measures, such as maintaining proper ventilator settings and providing close monitoring during oxygen therapy. This case also highlights the importance of developing guidelines and foolproof systems to ensure patient safety and improve postoperative outcomes by quickly and effectively managing potential complications and preventing accidents that may arise from essential and routine care.

## 4. Conclusion

This case report describes a rare complication of pneumothorax, pneumoperitoneum, and subcutaneous emphysema following inadvertent delivery of dry oxygen to a patient with a tracheostomy. This emphasizes the critical importance of proper airway humidification and adherence to established care protocols for patients with tracheostomy. The successful conservative management of these complications demonstrates that noninvasive approaches can be effective in carefully selected cases, even with extensive air dissection into multiple compartments.

This case serves as a powerful reminder of the potential consequences of seemingly minor deviations from standard care practices. This underscores the need for continued education, clear communication, and vigilance when caring for patients with complex airway disorders. By sharing these experiences, we hope to improve patient safety and prevent similar incidents.

Future research should focus on developing foolproof systems for airway management in postanesthesia care units and explore the factors that contribute to the successful conservative management of iatrogenic pneumothorax and related complications. Additionally, long-term follow-up studies of patients who experience such complications could provide valuable insights into potential late sequelae and inform best practices for ongoing care.

## Author contributions

**Conceptualization:** Jeong-Hee Kim.

**Validation:** Jeong-Hee Kim.

**Writing** – **original draft:** Jiyoung Kim.

**Writing** – **review & editing:** Hyeokjae Kwon.
